# 

**Published:** 1893-03

**Authors:** 


### EDITORIAL DEPARTMENT.

F. E. DANIEL, M. D., Editor.
S. E. HUDSON, M. D., Managing Editor.
A. J. SMITH. M. D., Galveston, Associate Editor.

EDITORIAL STAFF:

PROF. J. E. THOMPSON, M. D., Texas Medical College, Galveston; Surgery.
PROF. WM. KEILLER, M. D., Texas Medical College, Galveston; Obstetrics and Gynecology.
PROF. DAVID CERNA, M. D., Texas Medical College, Galveston; Therapeutics.
PROF. A. J. SMITH, M. D., Texas Medical College, Galveston; Medicine.
DR. ISADORE DYER, Tulane University; Dermatology.
DR. R. H. L. BIBB, Saltillo, Mexico; Foreign Correspondent.
DR. ROBT. MORRIS, Charity Hospital, N. O.; Clinical Reports.

———O———

The JOURNAL is the official organ of the Austin District Medical Society, the Wes Texas District Medical Society and the Galveston County Medical Society.

### ANNOUNCEMENT.

Dr. Daniel takes pleasure in announcing that Dr. S. E. Hudson has purchased a half interest in DANIEL'S TEXAS MEDICAL JOURNAL, and assumes the position of Managing Editor. All communications relating to advertisements and subscriptions may be addressed to him or to the JOURNAL, and he will receipt for all moneys due the JOURNAL.

Dr. Daniel commends Dr. Hudson to the profession, as a gentleman and a physician of high character and ability, and well worthy of their entire confidence and esteem. The name of the JOURNAL will remain unchanged.

